# Supplementary material for: Chromothripsis during telomere crisis is independent of NHEJ, and consistent with a replicative origin
Source: Genome Res. 2019 May;29(5):737–49. doi: 10.1101/gr.240705.118 (PMC6499312; doi:10.1101/gr.240705.118)
Supplement: Supplemental Material [file supp_gr.240705.118_Supplemental_file_1.zip › contigs/annotated_contigs/DB56/contig.3.DB56_length_377_mean_cov_12.5862068966.docx]

**DB56_length_377_mean_cov_12.5862068966**

```
CACCACCATCACCACCACCACCACCACCACCATCATCATCACCATCACCACTACCAT|T|TTTTGAGAGACAAAAAGCTGTATTTCCTG
 >chr10:35956413-35956470 + E=9e-11                        >chr2:3208495-3208716 + E=1e
CCATCAAGAGCCACAGAGCTGACAAGGGTGGCTTGATTTGGAGGACAGGAAAACTACAGAGAACACAGCACTGCGCTCACAGCAGACCC
-121
TACTACATGCAGGCGCACTCCCAGCAGACCCTACTACGTGTAGGCCGTGCTCACAGCAGACCCTACTACATGTAGGACTGTGCTCCCAG

CAGACCCTACTA|CT|CTTCCACCCTCTTATGTATAGATCCATACCAAATTGTTTAAATGCCCTGACTCTTCATCTGTAAAATAGGGCA
             >chr2:3129985-3130085 + E=1e-48
ATGACCGCCCACACTGCTCCTGCTG
```
